# The effects of aerobic exercises compared to conventional chest physiotherapy on pulmonary function, functional capacity, sputum culture, and quality of life in children and adolescents with cystic fibrosis: a study protocol for randomized controlled trial study

**DOI:** 10.1186/s13063-023-07719-w

**Published:** 2023-10-28

**Authors:** Nadia Hamedi, Mehrnaz Kajbafvala, Shabnam ShahAli, MohammadReza Pourahmadi, Alireza Eshghi, MohammadReza Modaresi Estahbanati

**Affiliations:** 1 Iranian Center of Excellence in Physiotherapy, Rehabilitation Research Center, Department of Physiotherapy, School of Rehabilitation Sciences, Iran University of Medical Sciences, Tehran, Iran; 2 Department of Paediatrics, School of Medicine, Ali-Asghar Children’s Hospital, Iran University of Medical Sciences, Tehran, Iran; 3 Department of Paediatrics, School of Medicine, Children’s Medical Center Hospital, Tehran University of Medical Sciences, Tehran, Iran

**Keywords:** Cystic fibrosis, Conventional chest physiotherapy, Aerobic exercise, Airway clearance

## Abstract

**Background:**

Cystic fibrosis (CF) is an autosomal recessive disorder caused by abnormal function of the chloride ion channels and characterized by pancreatic insufficiency and chronic endobronchial airway infection. Pulmonary dysfunction is very common and could lead to a reduction in the quality of life. Airway clearance techniques (ACT) and physical exercises are introduced as one of the main components of treatment. Therefore, it will be of interest to examine the effect of aerobic exercises compared to conventional chest physiotherapy (CPT) on pulmonary function, functional capacity, sputum culture, and quality of life in patients with CF.

**Methods:**

Thirty patients with CF will participate in a double-blind parallel controlled trial containing 18 sessions of treatment. Group A consists of CPT and placebo aerobic exercise, and group B includes aerobic exercise and placebo CPT. Pulmonary function, functional capacity, sputum culture, and quality of life will be evaluated with a spirometry test, 6-min walk test (6MWT), sputum culture test, and the Cystic Fibrosis Questionnaire-Revised (CFQ-R), respectively, before and after the intervention.

**Discussion:**

We will evaluate and compare the effectiveness of aerobic exercises and conventional chest physiotherapy on pulmonary function, functional capacity, sputum culture, and quality of life. Comparing these two treatment patterns can contribute to a better understanding of the effectiveness. Therefore, if there is a significant difference between the two treatments, the superior treatment will be prioritized clinically.

**Trial registration:**

https://www.irct.ir, IRCT20210505051181N5. Registered on 19 February 2023.

**Supplementary Information:**

The online version contains supplementary material available at 10.1186/s13063-023-07719-w.

## Introduction

### Background and rationale {6a and 6b}

Cystic fibrosis (CF) is one of the most common autosomal recessive diseases [1]. Pulmonary complications of CF are one of the most common causes of death with 60% mortality, especially in children and adolescents [1, 2]. Despite the lower prevalence of this disease in the Asian continent (1 per 35,000 births), the number of patients has been increasing over the last two decades [1]. The CF affects various systems with more involvement in the respiratory system, causing very viscous, hard, and sticky mucous secretions that accumulate in the excretory pathways [1]. This is the basis for inflammation, recurrent infections, and tissue fibrosis [3, 4]. With every episode of pulmonary infection, a high percentage of a patient’s lung function is irreversibly lost [4]. As a result, the quality of life is reduced and can be very disruptive in daily activities [1–3, 5]. Therefore, choosing the appropriate treatment is important in treating these patients and reducing the disease burden [3, 6]. Airway clearance techniques (ACT) and physical exercise are introduced as one of the main components of treatment [1, 3, 7, 8].

Conventional chest physiotherapy (CPT) is the oldest form of ACT that was introduced in the 1940s [9] and is recommended as the standard treatment that can be performed by all people of all ages [10]. Based on the results of most studies, including guidelines and systematic reviews, there is no meaningful difference between different ACTs (including CPT, active cycle of breathing technique (ACBT), autogenic drainage (AD), and oscillating positive expiratory pressure (OPEP) devices). They all lead to more clearance of the produced sputum, reducing the recurrence of pulmonary infections and slowing down the progression of lung destruction. This in turn improves lung function and increases the quality of life for patients [3, 7, 8, 11–16]. Choosing the best method depends on the patient’s ability to perform, preference, and available facilities [7].

Aerobic exercises are another important treatment option, as they increase endurance and cardiopulmonary fitness, aerobic capacity, mental health, and quality of life [17–19]. In addition, it is hypothesized that due to the increase in the respiratory rate and volumes and more passage of substances across the respiratory membranes and resultant increased ventilation, this treatment can also have significant effects on the evacuation of secretions [9, 20–23]. In addition to softening and moistening respiratory secretions, it also increases cough stimulation in patients and has been very effective in draining respiratory mucus [18, 20, 23, 24]. Limited studies also demonstrate its positive effects on respiratory infections [25, 26].

Hebestreit et al., in a randomized controlled trial study, investigated the effects of 1-year semi-supervised physical exercise [27]. The hypothesis was that an exercise program of 3 h per week can increase the pulmonary function level (forced expiratory volume in 1st second (FEV1)) of CF patients and its effects can be maintained for 6 months. The results demonstrated a significant FEV1 percent predicted increase in the control group, compared to the exercise group (2.70% predicted [95% CI, 0.13–5.26]; *P* = 0.04). Patients in the exercise group reported an increase in their level of daily activities (8.1% predicted [95% CI, 3.6–12.6]) and exercise capacity (4.5% predicted [95% CI, 1.0–8.0]). These effects were maintained for 1 year. Dweyer et al., in a randomized cross-over study, compared the effects of a treadmill, airway clearance (PEP), and control on sputum clearance and patient symptoms [18]. Based on the results, mucus clearance was higher in the PEP group (MD − 7%, 95% CI − 6 to − 8; *p* < 0.001), treadmill exercise (MD 3%, 95% CI 2–4; *p* < 0.001), and the control group, respectively. The number of coughs did not show a significant difference between the 3 groups, rather more apparent in the PEP group (MD 69, 95%CI 33–105; *p* < 0.01). Dweyer et al., in another cross-over study, compared the effects of treadmill exercise, and flutter on respiratory flow, characteristics of expectorated sputum, and clinical signs of subjects [28]. The maximum expiratory flow (MEF) in the treadmill (MD 1.68 ± 0.51, 95% CI;* p* < 0.01) and flutter (1.53 ± 0.25, 95% CI; *p* < 0.01) groups was higher. The mechanical impedance of the expectorated sputum of these two groups was not different. The number of coughs in the flutter group (MD 24 (18–34), 95% CI; *p* < 0.01), exercise (MD 4 (1–9), 95% CI; *p* < 0.01), and the control group were higher, respectively. Therefore, it was demonstrated conclusively that both treatments equally helped to clear the airways more effectively.

CPT is still the standard and most widely used method for children and adolescents with CF, because teaching other ACT methods has proven to be difficult for them to follow, and their effectiveness depends on correct learning and implementation processes. However, due to the inconvenience, time-consumption, and dependence of CPT on others, nowadays it is not uncommon, for most patients and even their families to prefer the alternative methods [7]. Also, aerobic exercise is a readily available activity that is practicable and clinically recommended [20].

Several studies have investigated the effectiveness and quality of the implementation of unsupervised interventions at home by patients or their parents [9, 29, 30], with results demonstrating that treatment adherence is 30% [9]. Study results reveal that unsupervised treatment has fewer positive effects compared with supervised treatment [25, 31, 32]. Numerous studies, including systematic reviews (alluding to the controversy in the results of aerobic exercises and the low methodological quality of these studies), have mentioned the need for further investigation in this field so that it can be credible to recommend the possibility of using aerobic exercises as a viable alternative and independent ACT [17, 20, 33–35].

To the best of the authors’ knowledge, a study comparing aerobic exercise with CPT with high-quality and precise design has not yet been investigated. Therefore, this study aims to compare the effect of aerobic exercises compared to CPT on pulmonary function, functional capacity, sputum culture, and quality of life in patients with CF. This is so that if significant changes are observed due to aerobic exercises compared to CPT, clinically, aerobic exercises can be recommended among treatment priorities.

### Objectives and hypotheses {7}

In this trial, we aim to include 30 children and adolescents of CF patients into a 1:1 randomized controlled trial study. We will compare the effects of conventional chest physiotherapy (CPT) and sham aerobic exercise in one group and aerobic exercise and sham CPT in the other group. The primary objective is to evaluate the effects of the 18-session aforementioned treatments on pulmonary function (FEV1) and functional capacity in children and adolescents with CF. The secondary objective is to assess the changes in sputum culture, pulmonary function (FVC), and quality of life of CF patients.

We hypothesize that there will be an increase by at least 7.1% in FEV1% percent predicted (minimally clinical important difference (MCID) considered for FEV1 [36]), a significant increase in the amount of FVC, at least 33-m increase in the distance covered in 6MWT (MCID considered for 6MWT [36]), a significant increase in the percentage of negative sputum culture tests and reduction of colony count tests after 6 weeks of aerobic exercise and CPT in CF patients, and a significant difference between the two groups. Also, the CFQ-R quality of life questionnaire score is expected to have a significant increase of at least 11.4 points in the physical domain, 7.3 points in the respiratory domain (MCIDs considered for CFQ-R [36]), and in the overall score.

### Trial design {8}

The current project is a double-blind 1:1 randomized controlled trial study designed to investigate and compare the effects of aerobic exercises and CPT in two parallel groups: group A consisting of CPT plus sham aerobic exercise and group B consisting of aerobic exercise and sham CPT. This study will be conducted at the CF specialized clinic of the Children’s Medical Center Hospital, Tehran, Iran; children and adolescents with confirmed diagnoses of CF will be randomly allocated to either group A or B.

Primary data points including FEV1, 6MWT, and secondary outcomes including sputum culture test (key secondary), FVC, and CFQ-R quality of life questionnaire will be assessed before and after treatments. It is expected that both treatments will have meaningful effects on the aforementioned variables and if significant changes are observed due to aerobic exercises compared to CPT, clinically, aerobic exercises will be recommended among treatment priorities.

## Methods: participants, interventions, and outcomes

### Study setting {9}

Data will be collected at the CF specialized clinic of the Children’s Medical Center Hospital, Tehran, Iran. Patients diagnosed with CF will be referred by a pediatric pulmonologist (MRME) or called among the previous files available.

### Eligibility criteria {10}

Table 1 summarizes the inclusion and exclusion criteria for the groups. Participants must meet all the eligibility criteria to be included.

**Table 1:** Inclusion and Exclusion criteria of the study

Inclusion criteria	Exclusion criteria
1- Confirmed diagnosis of cystic fibrosis based on positive sweat test or genetics test by a specialist doctor	1- Active hemoptysis, pneumothorax, hemodynamic instability, severe hypoxia, acute airway infection, and cognitive disorders
2- Age 6 to 18 years	2- Having cardiac disease such as heart failure or arrhythmia, neurologic and orthopedic disorders, or chest trauma
	3- History of fever, IV antibiotics, or hospitalization in the last 1 month
	4- Having severe uncontrolled gastroesophageal reflux
	5- Severe lung disease (FEV1% < 30%)
	6- Lung transplantation or on the waiting list
	7- Requirement of additional oxygen with exercise
	8- Having uncontrolled diabetics
	9- Improper patient cooperation during treatment sessions
	10- Absence in 3 or more consecutive sessions

The age range considered for inclusion criteria is 6–18. Based on the review and study of patient archives and registrations in the center, most of the patients who are referred to the CF specialized clinic are between the ages of 6 and 18, and this age range is the most accessible. In addition, those who are outpatients mostly have the same stage of chest disease; otherwise, patients with higher and more acute stages of chest disease are hospitalized and those with better conditions and in milder stages usually do not have routine clinical visits and are referred only for check-ups. Thus, participants will be among patients with more similar conditions.

### Who will take informed consent? {26a}, additional consent provisions for collection and use of participant data and biological specimens {26b}

The informed consent form will be created following the guidelines provided by the Ethics Committee of Iran University of Medical Sciences. The hospital’s secretary will be in charge of collecting the consent form from the patients, their parents, or caregivers. Before signing the consent form, all eligible patients or their parents will be fully informed about the interventions and possible adverse events. For more information regarding the consent form, please refer to Supplemental file 2.

### Interventions {11a}

The treatment session will commence 30 min after the first examination. Group A will first include the main treatment of CPT and then a placebo of aerobic exercise; group B will first include the main treatment of aerobic exercise and then a placebo of CPT (the inverse of Group A). The study will be conducted over 6 weeks, 3 times a week, with a total of 18 sessions. Make-up sessions will be allowed if a session is missed. The duration of the entire treatment session will last approximately 70 min.

### Group A (CPT and sham aerobic exercise)

At first, the participants will be placed in six standard postural drainage positions for a total of 30 min (Additional file 1: Supplemental Fig. 1A to F). Manual percussion and vibration will be performed on draining segments, in each position for 3–5 min. After completing the previous steps, the patient will be asked to sit down and cough for 1–2 min to expel the extracted secretions. In the following, in order to keep the patients blinded, the placebo aerobic exercise will be applied using a motorized stationary bike in two 15-min sections (Additional file 1: Supplemental Fig. 2). Between the two parts, 1–2 min will be allocated for rest. Throughout the exercise, the heart rate and percentage of arterial oxygen saturation (SpO2) of the patients will be monitored by a pulse oximeter. In this group, in order to eliminate the aerobic effects of the exercises, based on the method of previous studies [37], increasing the respiratory demands and breathing ventilation will be avoided until the end of the study. Therefore, the heart rate of the subject will not exceed 40% of HRmax during 30 min [38]. The total time of the exercises is 30 min, and at the end, 1–2 min will be given for coughing, if needed.

### Group B (aerobic exercise and sham CPT)

Progressive aerobic exercises will be conducted in two parts; the first will be 15 min on the treadmill and then a further 15 min on a stationary bike. Between the two sets, rest will be given for 1–2 min. A warm-up of 3 min with a gradual increase in speed will take place, and then 24 min of aerobic exercise with determined intensity (12 min on the treadmill and 12 min on a stationary bike), and finally a 3-min cool-down with a gradual decrease in speed will be conducted (Additional file 1: Supplemental Fig. 3A and B).

Throughout the training, the heart rate and SpO2 of patients will be monitored by a pulse oximeter. If there is a sharp drop in SpO2 below 85%, the heart rate is disproportionate to the conditions, and symptoms of severe shortness of breath or any other warning signs appear, the exercise will be stopped. During 24 min of aerobic exercises, the intensity of the exercise will be controlled through the heart rate of the patients (Table 2).

**Table 2:**
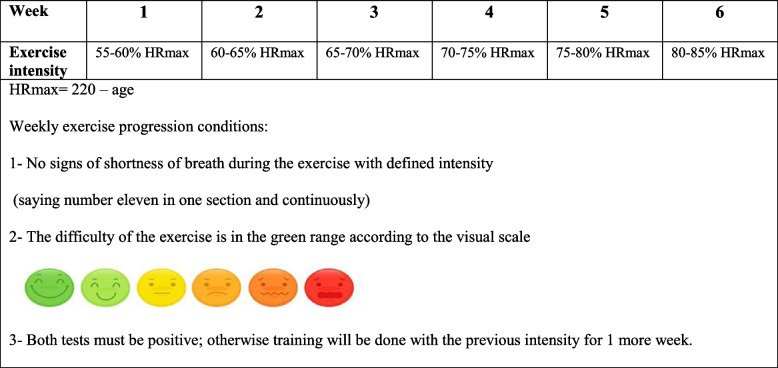
Progression of exercise intensity

In order to keep patients blinded, Sham CPT will be done by the experienced conductor (NH) exactly like the main CPT in group A, considering draining areas and hand placements. Modifications are made to the postural drainage positions and the applied manual force. To perform postural drainage, in order to remove the effect of gravity, only 2 positions of supine and prone without inclination will be used; the treated areas and procedures will be similar to group A. In order to eliminate the effect of manual percussion, these strikes will be done very gently with pressure just like touching the skin. In order to eliminate the effects of vibration and pressure, hands will be placed on the desired areas and no vibration or pressure will be applied during exhalation. The total duration will last approximately 30 min. At the end, 1–2 min will be allocated for any coughing, if needed.

### Criteria for discontinuing or modifying allocated interventions {11b}

The medical recommendations of individuals, such as drug treatments and airway clearance methods, will not be changed. Due to the risk of cross-infection, two infected patients will not be in the same environment at the same time [3]. Participants are asked to use their 7% sodium chloride nebulizer at home before their treatment session, to prepare the airways [39].

### Strategies to improve adherence to interventions {11c}

To improve patient motivation, the comprehensive rehabilitation protocol will be offered completely free of charge, ensuring a seamless connection between the patients and their dedicated team of healthcare professionals including the physiotherapist (NH) and physicians. This regular contact will enable patients to promptly communicate any symptoms they may experience. Participants will be given gifts like toys, edibles, and accessories for more encouragement and cooperation. These gifts will be of equal value and given to the patients at the end of the treatment session, in packages. Also, the treatment room has been well designed in colors and music will be accompanied, to provide a better environment for children. In addition, patient adherence to home ACT, ACBT, will be checked at the beginning of every session and marked in a provided checklist. Participants will be called adherent if at least 90% of the prescribed ACT is successfully performed.

### Relevant concomitant care permitted or prohibited during the trial {11d}

Before the treatment starts, the ACBT technique, one of the main ACTs proven to be effective and safe [11], will be thoroughly taught to the patients and their parents and a guidance paper will be given to them. One week before the treatment starts, patients are asked to replace their sports activities with ACBT. Also, they are asked to do the technique on the days between their treatment days, twice a day, at home. The ACBT implementation is checked by the conductor every session. The steps to implement the technique are breathing control, chest expansion, and forced expiration technique (FET) [11].

### Provisions for post-trial care {30}

There is no anticipated harm and compensation for trial participation and post-trial care is not applicable.

### Outcomes {12}

All outcomes will be measured on two measurement time points: at baseline, and after 6 weeks.

#### Primary outcomes

Pulmonary function (FEV1): FEV1 will be assessed objectively using a spirometry test (Additional file 1: Supplemental Fig. 4). After recording a maximum of 8 trials, the maximum values recorded in liters and the predicted percentage will be reported as results. The minimal clinically important difference (MCID) for FEV1% in CF has been reported as 7.1% [36].

Functional capacity: 6MWT will be performed based on the recommendations of the ATS Association [40]. A 22.5-m corridor, in an enclosed space and flat surface, is marked by two cones. Patients will be asked to walk as fast as they can within 6 min and cover the greatest possible distance between the two points without running. In the end, the distance covered in 6 min will be measured as the test result. The MCID for this test in patients with CF has been calculated to be 33 m [36].

#### Key secondary outcome

Sputum culture: The test method will be based on the study of Marguet et al. [41]. Two respiratory mucus sampling methods are used according to the patient’s ability (expectorated sputum/swab method). The obtained samples will be sent to the laboratory within 2 h. The main and common pathogens of CF like *Pseudomonas aeruginosa*, *Staphylococcus aureus*, and *Burkholderia cepecia* are cultured on the samples [42]. The culture test will be positive if pathogen culture is observed in the sample. In addition, in cases where the culture test is positive, the microbe colony count is also done and its amount is reported qualitatively (low/medium/high).

#### Secondary outcomes

Pulmonary function (FVC): FVC will be measured through a spirometry test, the same as FEV1.

Quality of life (CFQ-R): Quality of life will be subjectively assessed by the Persian version of the Cystic Fibrosis Questionnaire-revised (CFQ-R) [43, 44]. This questionnaire examines different domains (physical functioning, vitality, emotional state, social functioning, role functioning of the individual, body image, eating disorders, treatment burden, general perception of health, respiratory, and digestive symptoms) affecting the quality of life of the person in the last 2 weeks and has been prepared in 3 versions according to the age of the patient. The differing versions are for:

1. Children 6 to 13 years old
2. Parents of above-aged children
3. Adults 14 years old and older

The scale of answering questions is a 4-point scale. Total points are calculated and standardized in the range of 0 to 100. The closer the final score is to 100, the higher the quality of life. The MCID for the physical domain is 11.4 points increase, and for the respiratory domain, 7.3 has been calculated [36]. The validity and reliability of the Persian-translated version of the CFQ-R have been evaluated by Talebi et al. [43, 44]. The Cronbach-alpha for the CFQ-R was 0.65–0.91 for the children and parents versions, and ≥ 0.70 for the adult version, showing good internal consistency.

#### Other variables

In addition to the aforementioned outcomes, demographic and physiological variables such as age, sex, height, weight, medication history, physical activity level, and history of performing ACTs will be measured. These data will be reported as the baseline demographic information of the participants in Table 1 of the final paper, and they are balanced between the two groups by randomization method. There is no intention to do statistical analysis on these variables.

### Participant timeline {13}

**Fig. 1:**
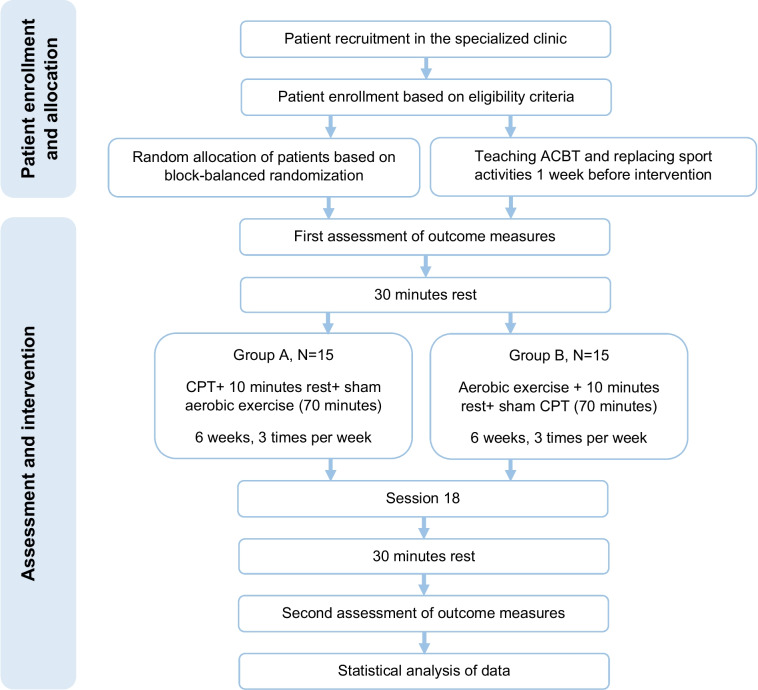
Patient timeline

Participant timeline is presented in Fig. 1 and study’s time schedule is presented in Table 3.

**Table 3:**
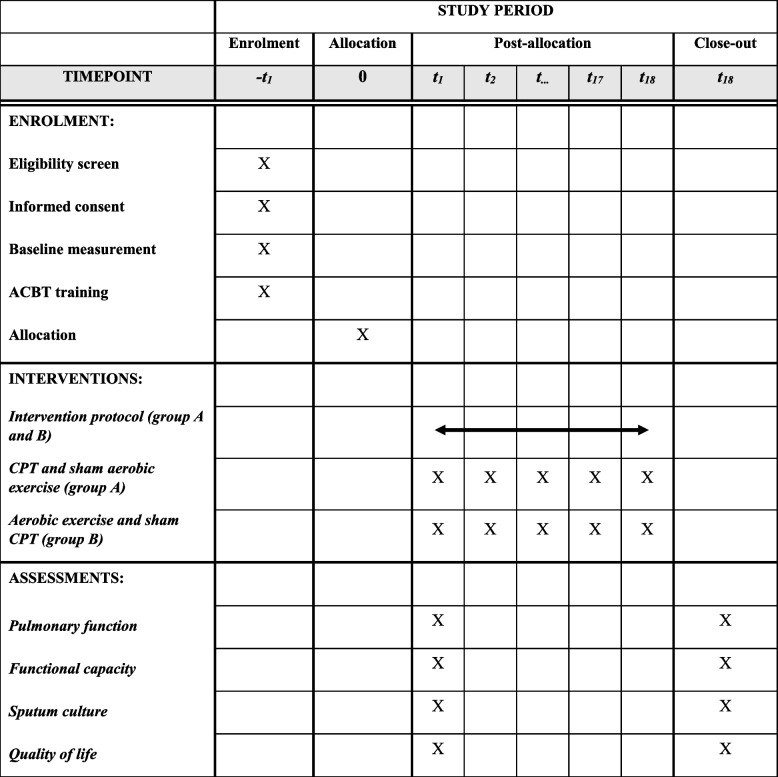
Study’s time schedule

### Sample size {14}

The prevalence of CF disease is lower in the Asian continent [1] as reported as 1 per 100,000–350,000 in the Middle East and 1 per 100,000 in Iran [5]. Thus, the number of diagnosed CF patients is low. On the other hand, the coordinating center has dedicated the CF specialized clinic of the Children’s Medical Center Hospital to the trial team, for a limited short time frame. Considering these factors, we selected the randomized controlled trial study by Sosa et al. [45] which was closest to our trial in terms of methodology and variables, as the basis for sample size calculation. In this study, 22 participants, 11 in each group, were recruited. Adding a 30% drop-out rate probability to each group, we anticipate recruiting 15 participants in each trial arm. Using power analysis, the required sample size with a power of 0.8 and an α of 0.05, a total sample size of 30 children and adolescents is determined.

### Recruitment {15}

In order to reach the target sample size, the pediatric pulmonologist from the CF specialized clinic will refer patients diagnosed with CF to the rehabilitation clinic of the Children’s Medical Center Hospital. The treatment course will be explained to the patients and their parents and they are encouraged to participate. Patients who are willing will enter the study based on the eligibility criteria. In addition, patients will be called among the previous files available at the center.

## Assignment of interventions: allocation

### Sequence generation {16a}

Participants will be randomly allocated to intervention groups A and B, in a 1:1 ratio, using block balanced randomization method.

### Allocation concealment {16b}

The randomization process was done by a statistician (MRP) before the start of the study. After the initial evaluation of participants by the examiner, who has enough experience and knowledge to do it, sealed numbered envelopes, corresponding to the sequential number of each person entered into the study, will be presented and the therapeutic intervention will be adjusted based on the letters inside the envelopes. The examiner and participants will be unaware of the letters inside the envelopes until the end of the study.

### Implementation {16c}

Treatment will be administered by a physical therapist who has sufficient experience and knowledge to perform it (NH).

## Assignment of interventions: blinding

### Who will be blinded {17a}

This study will be conducted as a double-blinded study. The patients, outcome assessor, and the statistician will be provided with information about the treatment process. However, they will remain unaware of the specific allocation throughout the study.

### Procedure for unblinding if needed {17b}

Throughout the study, there will be no permission to reveal the allocation to either the outcome assessor or the patients. However, once the study is completed, the patients will be informed about their group allocation.

## Data collection and management

### Plans for assessment and collection of outcomes {18a}

Outcomes are assessed at two time points, at baseline, and after treatment sessions, by the blinded examiner who has enough experience and knowledge to do it. Also, the demographic data will be gathered by the examiner in the first session. The Persian version of the CFQ-R questionnaire used for quality of life assessment has been validated by Talebi et al. and the Cronbach-alpha for the CFQ-R was 0.65–0.91 for children and parents’ version, and ≥ 0.70 for the adult version, showing good internal consistency [43, 44]. The questionnaire forms can be accessed through the Supplementary file 3.

### Plans to promote participant retention and complete follow-up {18b}

Participant retention and complete follow-up are promoted by the monitoring of the physical therapist. In case of withdrawal of patients from the study, their data will be counted as drop-outs.

### Data management {19}

Throughout all stages of the trial, utmost care will be taken to ensure the security and confidentiality of participants’ personal information. All participant data, including reports, data collection, process details, and administrative forms, will be stored in lockable file cabinets located in restricted-access areas. These documents will be identified solely by a coded ID number, maintaining anonymity. Instead of using names, the participant’s unique number identifier will be utilized in all data records. Forms, lists, logbooks, appointment books, and any other materials connecting participant ID numbers to identifiable information will be kept in a closed, separate file within a restricted zone. Only the primary investigator will have knowledge of the participants’ names associated with their respective numbers. To enhance data management, all collected data will be converted into electronic format and securely stored in password-protected files at the main study centers where the data originated. Access to this data will be strictly limited to the research team. Personal data will be stored separately from other information, treated as highly confidential, and accessible only to specifically authorized research team members. It will not be shared with any external parties, ensuring its complete privacy.

### Confidentiality {27}

The data of all patients will be stored and archived in a strictly confidential manner throughout all stages of the trial. The confidentiality of the data will be safeguarded not only during the trial but also after its completion.

### Plans for collection, laboratory evaluation, and storage of biological specimens for genetic or molecular analysis in this trial/future use {33}

Respiratory sputum samples will be obtained for the sputum culture test in the hospital’s laboratory. Patients will be provided with enough information about the test and sampling methods in the informed consent form (Supplemental file 1) alongside other information provided. There is no intention to do other analyses on these samples in this study; however, if they are used in future research, they will be reported and published as “secondary data analysis.”

## Statistical methods

### Statistical methods for primary and secondary outcomes {20a}

Data will be analyzed using Stata version 16. The normal distribution of data will be analyzed by the Shapiro–Wilk test. If the distribution is normal, parametric tests will be used for analysis before and after the intervention. To determine the homogeneity of variances (i.e., variances approximately equal across groups), Levene’s test will be employed. If the variance is equal, the analysis of covariance (ANCOVA) will be used. The covariance variable in this test will be the initial data values before the intervention. The significance level of the tests is set at 0.05. In addition to the significance index, the effect size index is also used by Cohen’s *d* method to compare two treatment methods for each variable. Therefore, the effect of the intervention on each of the dependent variables is determined, regardless of the sample size. According to the new interpretation presented, the effect size can be divided and interpreted as follows: from 0.01 to 0.2, very small; from 0.2 to 0.5, small; from 0.5 to 0.8, moderate; from 0.8 to 1.2, large; from 1.2 to 2, very large; and more than 2, very large [46].

### Interim analyses {21b}

Given that the designed interventions are non-invasive, cost-free, and not considered to be expensive, interim analyses are not deemed necessary. Additionally, the sample size has been predetermined in advance.

### Methods for any additional analyses {20b}

Our study does not plan to conduct any additional analyses beyond the intended outcomes. The statistical methods used to analyze the outcomes are clearly mentioned in the text. If any additional or subgroup analysis of variables or confounding factors becomes necessary during the study, it will be justified in the final paper and reported in a section titled “Secondary data analysis.”

### Methods in analysis to handle protocol non-adherence and any statistical methods to handle missing data {20c}

In order to increase adherence, patients will be contacted weekly and given gifts like toys, edibles, and accessories for more encouragement and cooperation. Treatment will be done in a well-designed room and accompanied by music. Missing data will be disposed of with the Multiple Imputation (MI) method.

### Plans to give access to the full protocol, participant-level data, and statistical code {31c}

To obtain access to the complete protocol, participant-level dataset, and STATA statistical codes for public use, interested parties must send a formal reasonable email request to the study’s corresponding author (MK). This is a necessary requirement for eligibility.

## Oversight and monitoring

### Composition of the coordinating center and trial steering committee {5d}

The coordinating center in this study is the specialized lung department in the Children’s Medical Center Hospital and its secretary contacts CF patients daily and arranges appointments. The project management group of the trial consists of the conductor (NH), the corresponding author (MK), and other authors of this study (SS, MRP, AE, MRME). The team will be in daily contact through social media and will monitor study conduct and data collection. The trial steering committee includes members of the physical therapy department of the Iran University of Medical Sciences and two lung specialists at the hospital. The meetings of this committee will be held once or twice a month and the conduct of the study and data collection process will be reported and analyzed in the form of slide presentation.

Stakeholders of this trial are CF patients, their families, and health care providers and their comments and suggestions will be gathered in a feasibility study, and if they are helpful with the improvement of the design, they will be implemented; accordingly, if any modifications to the study seem necessary, they will be reported and justified as amendments on IRCT.ir and in the final paper.

### Composition of the data monitoring committee, its role and reporting structure {21a}

The data monitoring committee consists of two of the authors (MK and MRP), a lung specialist doctor (MRME), and the statistician who will double check and analyze data and documents systematically, every week after it is entered into the STATA software by the conductor (NH).

### Adverse event reporting and harms {22}

At the end of the study, a questionnaire will be completed by patients or their parents to evaluate any unwanted side effects that may have occurred during the study and are reported in the order of prevalence. The adverse events most frequently reported are summarized in Table 4 and the adverse events questionnaire is provided in Supplemental file 4.

**Table 4:** Adverse events

Adverse events	Number	Percentage per total treatment
Shortness of breath and difficulty breathing		
Aggravation of coughs		
Pain in chest and rib cage		
Weakness and tiredness		
Gastroesophageal reflux		
Muscle soreness in lower limbs		
Nausea		
Dizziness		

### Frequency and plans for auditing trial conduct {23}

The Ethics Committee has been accepted on IRCT.ir and the trial conduct information will be updated every month on the mentioned platform. If any changes are made to the study, they will be monitored by the ethics committee on IRCT. The data monitoring committee (MK, MRP, MRME, and statistician) will check and analyze data and documents systematically, every week. Also, the trial steering committee will have meetings once or twice a month.

### Protocol amendments {25}

Any modifications to the study protocol require approval from the Ethical Committee. If changes are needed based on feedback from patients, stakeholders, or other reasons, they will be discussed and justified on IRCT.ir and in a separate section of the final paper.

### Dissemination policy {31a}

Upon the completion of the study, all collected data will undergo statistical analysis and subsequently be published in international, peer-reviewed, high-impact factor, academic journals. Additionally, the findings will be presented at national and international CF-related conferences. Participants will be allowed to obtain a copy of the scientific publication(s) containing the complete, de-identified dataset.

## Discussion

The present study will investigate the effect of aerobic exercises compared to conventional chest physiotherapy on pulmonary function, functional capacity, sputum culture, and quality of life in patients with Cystic Fibrosis.

Based on previous studies, respiratory problems, are the most common and important symptoms following CF [1]. Some investigations reported a reduction in patients’ quality of life and spending a lot of time and money on treatment because of respiratory problems [1, 3]. Other findings revealed positive effects of various types of ACTs on patients’ pulmonary function [7, 11–15]. In a comparison between vigorous physical exercises and maintaining the previous physical activity level as a control, after 6 months of partially supervised training sessions, a significant increase in exercise capacity was observed [27]. Additionally, based on the results of systematic reviews, because of the low quality of the studies, no definite opinion has been made about the effects of physical exercises [7, 35]. However, there is a lack of studies that evaluated the effect of exercises on pulmonary function and quality of life, and the most positive effects of exercises have been seen on maximum aerobic capacity [35].

To the best of the author’s knowledge, the studies conducted in the field of physical exercises are very limited, which indicates a need for more studies with higher methodological quality to be carried out in order to facilitate a commentary on the effect of physical exercises as ACT. In addition, no studies have evaluated the effects of supervised aerobic exercises compared to conventional chest physiotherapy on the most important health indicators of CF patients, including FEV1.

The main result of this study will explore and discuss the question of what is the effect of supervised aerobic exercises on pulmonary function, functional capacity, sputum culture, and quality of life? If there is a significant difference between the two treatments, one of them can be prioritized clinically.

The strength of our study is the measurement of pulmonary function and quality of life. The study will be a double-blinded RCT and performed under the complete supervision of the physiotherapist. The duration of the treatment is 6 weeks, which is suitable for measuring the medium-term effects of the treatment. Patients’ aerobic exercises will be as progressive as possible according to their condition. Among other strengths of this study is the use of multiple clinical, functional, laboratory, and self-report variables at the same time so that treatment results can be discussed in a wider dimension.

### Delimitations and limitations

Our study will have some delimitations and limitations. The age range considered as inclusion criteria is 6–18. Based on the review and study of patient archives and registrations in the center, most of the patients who are referred to the CF specialized clinic are between the ages of 6 and 18, and this age range is the most accessible. Therefore, the obtained data can only be generalized with patients of the same age range and it will not apply to younger kids and adults. Future studies can consider other age ranges for more generalizability. Due to the lower prevalence of CF disease in Iran, the number of patients with a definite diagnosis of the disease is low. As a result, the sample size calculated for this study was small. Future research could be done by recruiting more participants. On the other hand, the coordinating center has dedicated the CF specialized clinic of the Children’s Medical Center Hospital to the trial team, for a limited short time frame. So there is no intention to have a long-term follow-up of participants. In addition, this study has been designed as a medium-term study, and treatment duration will be 6 weeks. More research could be done considering long-term treatment periods and long-term follow-up.

## Trial status

The current protocol version is version 1 from July 23, 2023. Recruitment and enrolment of participants started on July 23, 2023. The expected recruitment end date is January 20, 2024, when the planned sample size is achieved.

### Supplementary Information

**Additional file 1: Figure s1.** 6 Postural drainage positions. **Figure s2.** Motorized stationary bike. **Figure s3.** Aerobic exercise. **Figure s4.** Spirometry test.

**Additional file 2.**

**Additional file 3.**

**Additional file 4.**

**Additional file 5.**

## Data Availability

The final trial dataset will be accessible by sending a justifiable email to the corresponding author (MK).

## Abbreviations

CF: Cystic fibrosis
ACT: Airway clearance technique
CPT: Conventional chest physiotherapy
6MWT: 6-Minute walk test
CFQ-R: Cystic fibrosis questionnaire- revised
ACBT: Active cycle of breathing technique
OPEP: Oscillating positive expiratory pressure
FEV1: Forced expiratory volume in 1st second
FVC: Forced vital capacity
PEP: Positive expiratory pressure
MCID: Minimally clinical important difference
FET: Forced expiration technique
MI: Multiple Imputation
HRmax: Maximum heart rate

## References

[1] Bell SC, Mall MA, Gutierrez H, Macek M, Madge S, Davies JC (2020). The future of cystic fibrosis care: a global perspective. Lancet Respir Med.

[2] Dickinson KM, Collaco JM (2021). Cystic Fibrosis. Pediatr Rev.

[3] National Guideline A (2017). National Institute for Health and Care Excellence: Guidelines. Cystic Fibrosis: Diagnosis and management.

[4] Taylor SL, Leong LEX, Ivey KL, Wesselingh S, Grimwood K, Wainwright CE (2020). Total bacterial load, inflammation, and structural lung disease in paediatric cystic fibrosis. J Cyst Fibros.

[5] Mirtajani S, Farnia P, Hassanzad M, Ghanavi J, Farnia P, Velayati A (2017). Geographical distribution of cystic fibrosis; the past 70 years of data analyzis. Biomed Biotechnol Res J (BBRJ).

[6] GBD 2019 Diseases and Injuries Collaborators. Global burden of 369 diseases and injuries in 204 countries and territories, 1990–2019: a systematic analysis for the Global Burden of Disease Study 2019. Lancet. 2020;396(10258):1204–22. 10.1016/S0140-6736(20)30925-9 PMC7567026 33069326

[7] Wilson LM, Morrison L, Robinson KA (2019). Airway clearance techniques for Cystic Fibrosis: an overview of Cochrane systematic reviews. Cochrane Database Syst Rev.

[8] Button BM, Wilson C, Dentice R, Cox NS, Middleton A, Tannenbaum E (2016). Physiotherapy for cystic fibrosis in Australia and New Zealand: a clinical practice guideline. Respirology (Carlton, Vic).

[9] Rowbotham NJ, Daniels TE (2022). Airway clearance and exercise for people with cystic fibrosis: balancing longevity with life. Pediatr Pulmonol.

[10] Marks JH (2007). Airway clearance devices in cystic fibrosis. Paediatr Respir Rev.

[11] McKoy NA, Wilson LM, Saldanha IJ, Odelola OA, Robinson KA (2016). Active cycle of breathing technique for cystic fibrosis. Cochrane Database Syst Rev.

[12] McIlwaine M, Button B, Nevitt SJ (2019). Positive expiratory pressure physiotherapy for airway clearance in people with cystic fibrosis. Cochrane Database Syst Rev.

[13] Morrison L, Milroy S (2020). Oscillating devices for airway clearance in people with cystic fibrosis. Cochrane Database Syst Rev.

[14] Rocamora-Pérez P, Benzo-Iglesias MJ, Valverde-Martínez M, García-Luengo AV, Aguilar-Parra JM, Trigueros R (2022). Effectiveness of positive expiratory pressure on patients over 16 years of age with cystic fibrosis: systematic review and meta-analysis. Ther Adv Respir Dis.

[15] Warnock L, Gates A (2015). Chest physiotherapy compared to no chest physiotherapy for cystic fibrosis. Cochrane Database Syst Rev.

[16] Athanazio RA, da Silva FLVRF, Vergara AA, Ribeiro AF, Riedi CA, Procianoy EFA (2017). Brazilian guidelines for the diagnosis and treatment of cystic fibrosis. J Bras Pneumol.

[17] Ward N, Stiller K, Holland AE (2019). Exercise as a therapeutic intervention for people with cystic fibrosis. Expert Rev Respir Med.

[18] Dwyer TJ, Daviskas E, Zainuldin R, Verschuer J, Eberl S, Bye PTP (2019). Effects of exercise and airway clearance (positive expiratory pressure) on mucus clearance in Cystic Fibrosis: a randomised crossover trial. Eur Respir J.

[19] Hebestreit H, Kriemler S, Radtke T (2015). Exercise for all cystic fibrosis patients: is the evidence strengthening?. Curr Opin Pulm Med.

[20] Gruet M, Saynor ZL, Urquhart DS, Radtke T. Rethinking physical exercise training in the modern era of cystic fibrosis: A step towards optimizing short-term efficacy and long-term engagement. J Cyst Fibros. 2022;21(2):e83–98. 10.1016/j.jcf.2021.08.004 34493444

[21] Smith LJ, Marshall H, Bray J, Wildman M, West N, Horsley A (2021). The effect of acute maximal exercise on the regional distribution of ventilation using ventilation MRI in CF. J Cyst Fibros.

[22] Puppo H, Torres-Castro R, Vasconcello-Castillo L, Acosta-Dighero R, Sepúlveda-Cáceres N, Quiroga-Marabolí P (2020). Physical activity in children and adolescents with cystic fibrosis: a systematic review and meta-analysis. Pediatr Pulmonol.

[23] Kriemler S, Kieser S, Junge S, Ballmann M, Hebestreit A, Schindler C (2013). Effect of supervised training on FEV1 in cystic fibrosis: a randomised controlled trial. J Cyst Fibros.

[24] Dwyer TJ, Alison JA, McKeough ZJ, Daviskas E, Bye PTP (2011). Effects of exercise on respiratory flow and sputum properties in patients with cystic fibrosis. Chest.

[25] Urquhart D, Sell Z, Dhouieb E, Bell G, Oliver S, Black R (2012). Effects of a supervised, outpatient exercise and physiotherapy programme in children with cystic fibrosis. Pediatr Pulmonol.

[26] Moeller A, Stämpfli SF, Rueckert B, Rechsteiner T, Hamacher J, Wildhaber JH (2010). Effects of a short-term rehabilitation program on airway inflammation in children with cystic fibrosis. Pediatr Pulmonol.

[27] Hebestreit H, Kriemler S, Schindler C, Stein L, Karila C, Urquhart DS (2022). Effects of a partially supervised conditioning program in cystic fibrosis: an international multicenter, randomized controlled trial (ACTIVATE-CF). Am J Respir Crit Care Med.

[28] Dwyer TJ, Zainuldin R, Daviskas E, Bye PT, Alison JA (2017). Effects of treadmill exercise versus Flutter® on respiratory flow and sputum properties in adults with Cystic Fibrosis: a randomised, controlled, cross-over trial. BMC Pulm Med.

[29] Downs JA, Roberts CM, Blackmore AM, Le Souëf PN, Jenkins SC (2006). Benefits of an education programme on the self-management of aerosol and airway clearance treatments for children with cystic fibrosis. Chron Respir Dis.

[30] Schmidt AM, Jacobsen U, Bregnballe V, Olesen HV, Ingemann-Hansen T, Thastum M (2011). Exercise and quality of life in patients with cystic fibrosis: a 12-week intervention study. Physiother Theory Pract.

[31] Rovedder PM, Flores J, Ziegler B, Casarotto F, Jaques P, Barreto SS (2014). Exercise programme in patients with Cystic Fibrosis: a randomized controlled trial. Respir Med.

[32] Brivio A, Orenti A, Barbisan M, Buonpensiero P, Ros M, Gambazza S. Home physiotherapists assisting follow-up treatment in cystic fibrosis: a multicenter observational study. Monaldi Arch Chest Dis. 2021;91(2). 10.4081/monaldi.2021.1619. 10.4081/monaldi.2021.1619 33926178

[33] Bradley JM, Moran FM, Stuart EJ (2006). Evidence for physical therapies (airway clearance and physical training) in cystic fibrosis: an overview of five Cochrane systematic reviews. Respir Med.

[34] Elbasan B, Tunali N, Duzgun I, Ozcelik U (2012). Effects of chest physiotherapy and aerobic exercise training on physical fitness in young children with Cystic Fibrosis. Ital J Pediatr.

[35] Radtke T, Smith S, Nevitt SJ, Hebestreit H, Kriemler S (2022). Physical activity and exercise training in cystic fibrosis. Cochrane Database Syst Rev.

[36] Bhatia R, Kaye M, Roberti-Miller A (2020). Longitudinal assessment of exercise capacity and quality of life outcome measures in cystic fibrosis: a year-long prospective pilot study. J Eval Clin Pract.

[37] Lee MJ, Kilbreath SL, Singh MF, Zeman B, Lord SR, Raymond J (2008). Comparison of effect of aerobic cycle training and progressive resistance training on walking ability after stroke: a randomized sham exercise-controlled study. J Am Geriatr Soc.

[38] Kisner C, Colby L, Borstad J. Therapeutic Exercise: Foundations and Techniques. McGraw Hill. 2018. p. 7e. https://fadavispt.mhmedical.com/content.aspx?bookid=2262§ionid=175446320. Accessed 25 Oct 2023.

[39] Elkins MR, Bye PT (2011). Mechanisms and applications of hypertonic saline. Journal of the Royal Society of Medicine..

[40] ATS Committee on Proficiency Standards for Clinical Pulmonary Function Laboratories. ATS statement: guidelines for the six-minute walk test. Am J Respir Crit Care Med. 2002;166(1):111–7. 10.1164/ajrccm.166.1.at1102 12091180

[41] Marguet C, Houdouin V, Pin I, Reix P, Huet F, Mittaine M (2021). Chest physiotherapy enhances detection of Pseudomonas aeruginosa in nonexpectorating children with Cystic Fibrosis. ERJ Open Res..

[42] Eshghi A, Khanbabaee G, Hassanzad M, Tabatabaee SA, Rezaei M (2019). Evaluation of fraction of exhaled nitric oxide (FeNO) in CF children and its association with sputum culture. J Compr Ped..

[43] Talebi S, Sayedi SJ, Ranjbar G, Khadem Rezaeian M, Bbarghchi H, Kazemi Sefat G (2021). Towards the Validation of the Persian Version of the Revised Cystic Fibrosis Quality of Life Questionnaire for children and parents (CFQ-R). Int J Pediatr.

[44] Talebi S, Sayedi SJ, Ranjbar G, Khadem Rezaeian M, Barghchi H, Safarian M (2022). Towards the validation of the Persian translation of the revised cystic fibrosis quality of life questionnaire in adolescents and adults (CFQ-R 14+). Int J Pediatr.

[45] Santana Sosa E, Groeneveld IF, Gonzalez-Saiz L, López-Mojares LM, Villa-Asensi JR, Barrio Gonzalez MI (2012). Intrahospital weight and aerobic training in children with cystic fibrosis: a randomized controlled trial. Med Sci Sports Exerc.

[46] Sawilowsky SS (2009). New effect size rules of thumb. J Mod Appl Stat Methods.

