# Supplementary material for: The effects of aerobic exercises compared to conventional chest physiotherapy on pulmonary function, functional capacity, sputum culture, and quality of life in children and adolescents with cystic fibrosis: a study protocol for randomized controlled trial study
Source: Trials. 2023 Oct 28;24:695. doi: 10.1186/s13063-023-07719-w (PMC10612191; doi:10.1186/s13063-023-07719-w)
Supplement: Supplementary file 1 — Additional file 1: Figure s1. 6 Postural drainage positions. Figure s2. Motorized stationary bike. Figure s3. Aerobic exercise. Figure s4. Spirometry test. [file 13063_2023_7719_MOESM1_ESM.docx]

**Appendix**
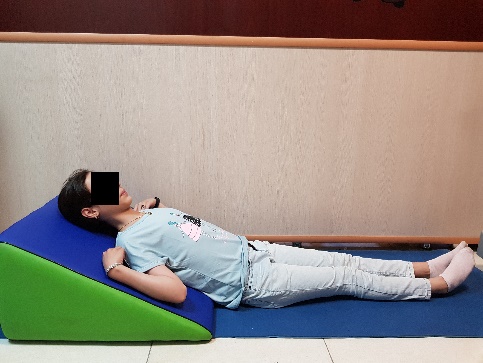

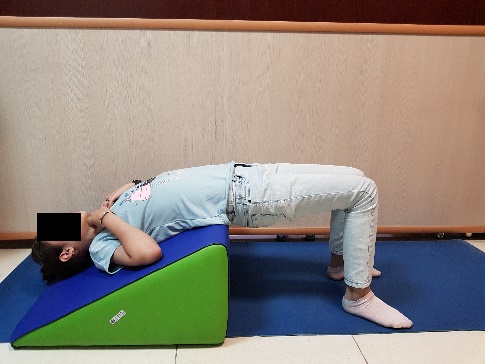

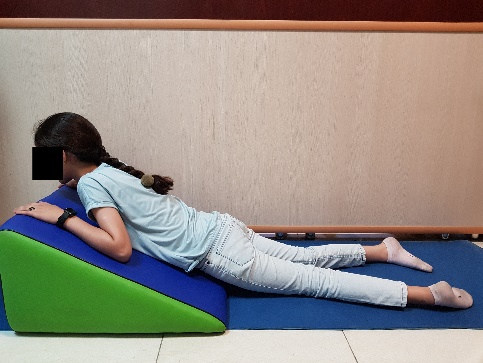


B

C

A

**
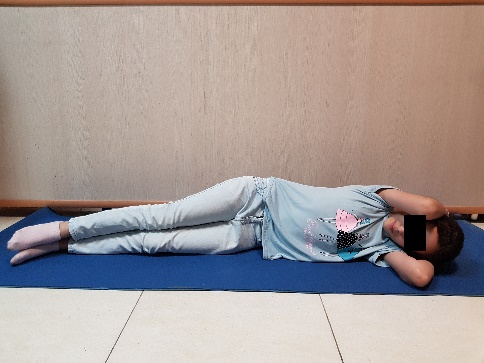

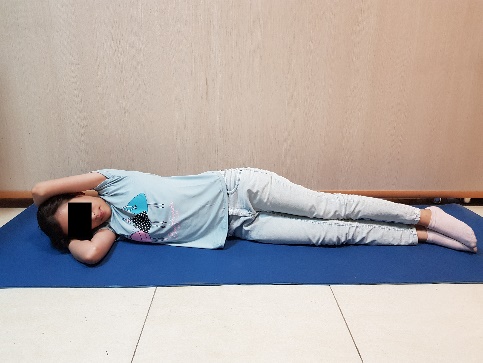

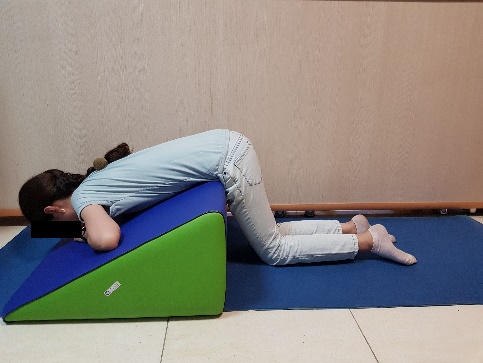
 Figure s1**. 6 postural drainage positions

F

E

D

**
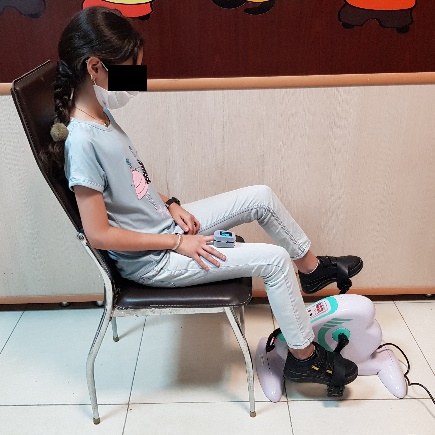
**

**Figure s2.** Motorized stationary bike


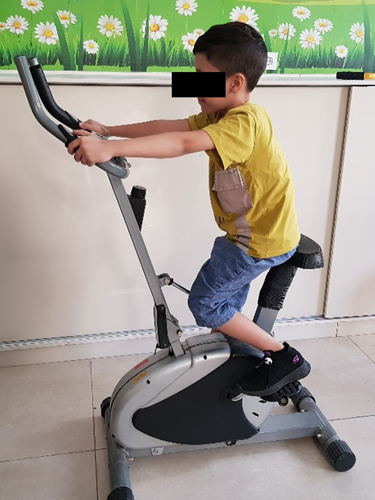


A

B

**
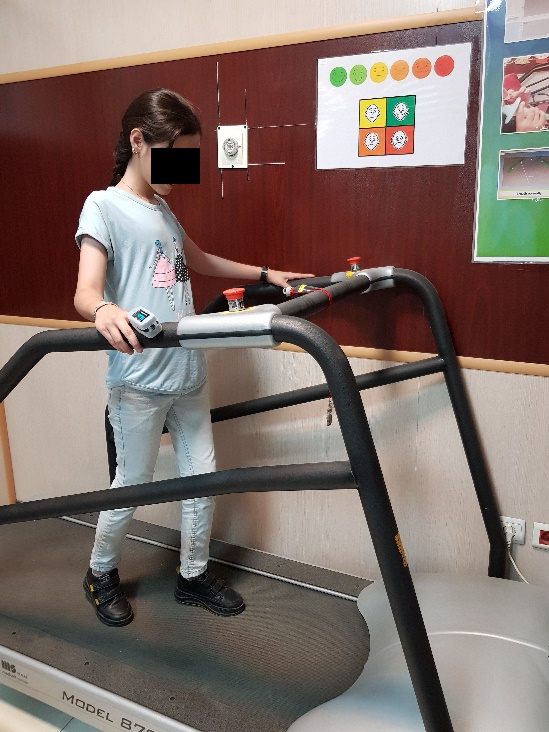
Figure s3.** Aerobic exercise


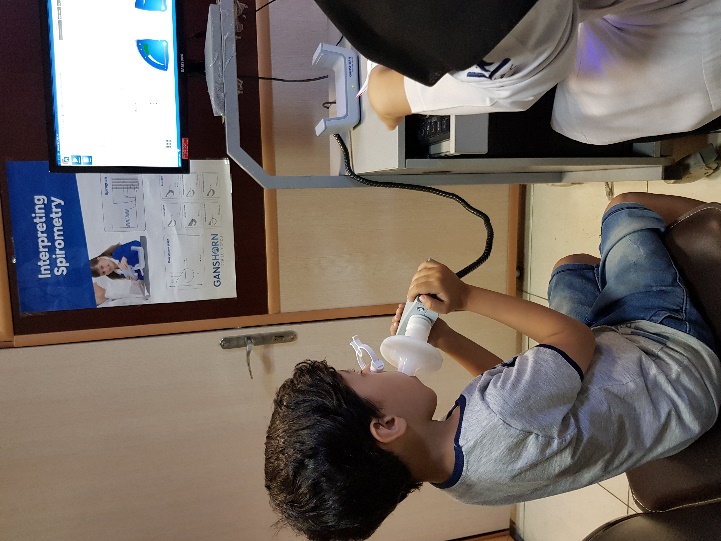


**Figure s4.** Spirometry test
