# Supplementary material for: The effects of aerobic exercises compared to conventional chest physiotherapy on pulmonary function, functional capacity, sputum culture, and quality of life in children and adolescents with cystic fibrosis: a study protocol for randomized controlled trial study
Source: Trials. 2023 Oct 28;24:695. doi: 10.1186/s13063-023-07719-w (PMC10612191; doi:10.1186/s13063-023-07719-w)
Supplement: Supplementary file 2 — Additional file 2. [file 13063_2023_7719_MOESM2_ESM.pdf]

رضایت نامه شرکت در طرح

« تاثیر تمرینات هوازی در مقایسه با فیزیوتراپی رایج قفسه سینه بر عملکرد ریوی، ظرفیت تمرین، ظرفیت عملکردی، کشت خلط و کیفیت زندگی در بیماران مبتلا به فیبروز کیستیک: یک کارآزمایی بالینی شاهددار تصادفی شده »

آقای/ خانم محترم ....

بدین وسیله از شما جهت شرکت در پژوهش فوق‌الذکر دعوت به عمل می‌آید. اطلاعات مربوط به این پژوهش در این برگه خدمتتان ارائه شده است و شما برای شرکت یا عدم شرکت در این پژوهش آزاد هستید.

شما مجبور به تصمیم گیری فوری نیستید و برای تصمیم گیری در این باره می‌توانید سوالات خود را از تیم پژوهشی بپرسید و با هر فردی که مایل باشید مشورت نمایید. قبل از امضای این رضایت نامه مطمئن شوید که متوجه تمامی اطلاعات این فرم شده‌اید و به تمام سوالات شما پاسخ داده شده است.

مجری پژوهش (نادیا حامدی)، استاد راهنمای پژوهش (دکتر کجباف والا)

۱. من می‌دانم که اهداف این پژوهش عبارتند از:

بررسی و مقایسه تاثیری که تمرینات هوازی و فیزیوتراپی تنفسی بر سلامت ریه، فعالیت فیزیکی، عفونت تنفسی و کیفیت زندگی بیماران مبتلا به فیبروز کیستیک دارد؛ با توجه به نقش مهمی که فیزیوتراپی در کمک به سیستم تنفسی این بیماران، با استفاده از روش های مستقل، آسان، کم هزینه و در دسترس دارد، در صورتی که نتایج این پژوهش اثرات مثبت تمرینات هوازی و فیزیوتراپی تنفسی را نشان دهد، می توان انجام این تمرینات را جزء اولویت های درمانی در نظر گرفت.

۲. من می‌دانم که شرکت من در این پژوهش کاملاً داوطلبانه است و مجبور به شرکت در این پژوهش نیستم.

به من اطمینان داده شد که اگر حاضر به شرکت در این پژوهش نباشم، از مراقبت‌های معمول تشخیصی و درمانی محروم نخواهم شد و رابطه درمانی من با مرکز درمانی و پزشک معالجم دچار اشکال نمی‌شود.

۳. من می‌دانم که حتی پس از موافقت با شــرکت در پژوهش می‌توانم هر وقت که بخواهم، پس از اطلاع به مجری، از پژوهش خارج شوم و خروج من از پژوهش باعث محرومیت از دریافت خدمات درمانی معمول برای من نخواهد شد.

۴. نحوه‌ی همکاری اینجانب در این پژوهش به این‌صورت است:

در این مطالعه در یک گروه فیزیوتراپی تنفسی و دارونمای تمرینات هوازی، و در گروه دیگر تمرینات هوازی و دارونمای فیزیوتراپی تنفسی به عنوان درمان انجام می شود. اینجانب به شکل کاملا تصادفی، در یکی از دو گروه قرار می گیرم.

در این مطالعه اطلاعاتی از جمله نام و نام خانوادگی، آدرس، شماره تماس، سن، جنس، قد، وزن، سطح سواد، داروهای ریوی، سابقه انجام فیزیوتراپی های تنفسی و فعالیت های ورزشی، سابقه علائم تنفسی و بیماری های خاص، سابقه تشدید علائم در ۱ماه اخیر، سابقه دیابت و استفاده از داروی نبولایزر از من سوال می شود.

در این مطالعه تست کشت خلط، اسپیرومتری و تست ورزش، همچنین تست ۶ دقیقه راه رفتن و پرسشنامه کیفیت زندگی، ۲ بار، پیش از درمان و پس از اتمام آن، از من گرفته می شود.

مدت زمان این مطالعه از شروع جلسات درمان ۶ هفته، ۳ بار در هفته، ترجیحا ۱ روز در میان بوده و هر جلسه ۷۰ دقیقه طول می کشد. کل جلسات درمان ۱۸ جلسه می باشد.

در فاصله بین مراجعات، باید از شرکت در فعالیت های ورزشی خودداری کرده و تمرین ACBT را که به من آموزش داده شده است، روزی ۲ بار، در منزل انجام دهم. همچنین، پیش از مراجعه حضوری، از نبولایزر سدیم کلراید خود در خانه استفاده کنم. در توصیه ها و آموزش های پزشک متخصص، تغییری ایجاد نمی شود.

پیگیری وضعیت و تغییرات علائم من از طریق پیام و تماس تلفنی صورت می گیرد. هم چنین، در طی جلسات درمان، تغییرات علائم من تحت نظر می باشد.

۵. منافع احتمالی شرکت اینجانب در این مطالعه به این شرح است:

افزایش سطح سلامت، بهبودی علائم تنفسی، افزایش سطح فعالیت فیزیکی و ارتقاء زندگی روزمره

آموزش و یادگیری صحیح تمرین های فیزیوتراپی تحت نظر متخصص

دریافت رایگان خدمات تشخیصی و درمانی

کمک به ارتقاء روش های درمانی موجود برای فیبروز کیستیک

۶. آسیب‌ها و عوارض احتمالی شرکت در این مطالعه به این شرح است:

احتمال کم تا متوسط احساس بی قراری، درد، ناراحتی و دشواری تحمل درمان

احتمال متوسط تشدید سرفه ها و تنگی نفس یا احساس فشار در قفسه سینه

احتمال متوسط وقوع ریفلاکس (بازگشت محتویات معده به مری) و حالت تهوع و استفراغ در صورتیکه آخرین وعده غذایی کمتر از ۱ ساعت با جلسه درمان فاصله داشته باشد.

احتمال کم تا متوسط احساس سرگیجه، ضعف و خستگی، درد و کوفتگی در عضلات پاها

احتمال کم احساس تپش قلب و افت قند خون

۷. در صورت عدم تمایل به شرکت در مطالعه روش معمول درمانی برای من ارائه خواهد شد که منافع و عوارض آن به این شرح است:

در این پژوهش، با توجه به اینکه درمان ها تحت نظر متخصص انجام می شوند، به شکل اصولی اجرا شده و براساس پژوهش هایی که قبلا انجام شده اند، می تواند اثرات بسیار خوبی بر بهبودی علائم ریوی و افزایش سطح سلامت، داشته باشد. به علاوه، فرآیند تغییرات علائم، در طول مدت مطالعه، تحت نظر بوده و در صورت بروز عوارض احتمالی، به سرعت تشخیص و پیگیری می شود و این عمل، رایگان خواهد بود. هم چنین، آموزش تمرین های فیزیوتراپی به شکل اصولی آن انجام شده و اجرای درست آن، توسط متخصص پیگیری می شود. روش های درمانی ای که در این پژوهش مورد بررسی قرار می گیرند، درمان های در دسترس و کم هزینه ای بوده و هر کدام از دو روش که اثر بهتری نشان بدهد، در زندگی روزمره نیز قابل انجام است.

درمان های معمول از جمله داروها و نبولایزرها، عوارض فیزیکی مثل ضعف و خستگی را ندارند و انجام آنها زمان کمتری نیاز دارد، اما به تنهایی در تخلیه خلط موثر نبوده و بر فعالیت فیزیکی نیز تاثیر چشمگیری ندارد. به علاوه،

در مواردی که تمرینات فیزیوتراپی، در منزل توسط بیمار یا والدین او انجام می شود، اغلب به دلیل آموزش ناصحیح و عدم نظارت، اشتباه یا ناکافی بوده و تاثیر چندانی ندارد. به همین دلیل، بیماران پیگیر انجام آن نیستند و این مسئله می تواند سلامت آنان را به خطر بیاندازد.

۸. من می‌دانم که دست اندر کاران این پژوهش، کلیه اطلاعات مربوط به من را نزد خود به صورت محرمانه نگه‌داشته و فقط اجازه دارند فقط نتایج کلی و گروهی این پژوهش را بدون ذکر نام و مشخصات اینجانب منتشر کنند.

۹. می‌دانم که کارگروه اخلاق در پژوهش با هدف نظارت بر رعایت حقوق اینجانب می‌تواند به اطلاعات من دسترسی داشته باشد.

۱۰. من می‌دانم که هیچ‌یک از هزینه‌های انجام مداخلات پژوهشی به شرح ذیل بر عهده من نخواهد بود:

هزینه تست های اسپیرومتری،کشت خلط و تست ورزش و وسایل مصرفی در آن ها

هزینه درمان های فیزیوتراپی شامل فیزیوتراپی تنفسی و تمرین هوازی

هزینه تشخیص و درمان هرگونه عوارض احتمالی مرتبط با پژوهش

هزینه تهیه نبولایزر و محلول سدیم کلراید

هزینه رفت و آمد به مرکز برای پژوهش

۱۱. پژوهشگر خانم نادیا حامدی و استاد راهنما دکتر مهرناز کجباف والا جهت پاسخگویی به اینجانب معرفی شد و به من گفته شد تا هر وقت مشکلی یا سوالی در رابطه با شرکت در پژوهش مذکور پیش آمد با ایشان در میان بگذارم و راهنمایی بخواهم.

آدرس و شماره تلفن همراه ایشان به شرح زیر به من ارائه شد:

آدرس: میرداماد، میدان مادر، خیابان شاه نظری، کوچه مددکاران، دانشکده توانبخشی، دپارتمان فیزیوتراپی

تلفن همراه پژوهشگر: ۰۹۳۹۸۱۳۰۱۳۲

تلفن ثابت پژوهشگر: ۰۲۶۳۴۴۵۱۰۲۷

۱۲. من می‌دانم که اگر در حین و بعد از انجام پژوهش هر مشکلی اعم از جسمی و روحی به علت شرکت در این پژوهش برای من پیش آمد درمان عوارض، و هزینه‌های آن و غرامت مربوطه بر عهده مجری خواهد بود.

۱۳. من می‌دانم اگر اشکال یا اعتراضی نسبت به دست اندرکاران یا روند پژوهش دارم می‌توانم با کارگروه اخلاق در پژوهش دانشگاه علوم پزشکی ایران به آدرس: تهران، دانشگاه علوم پزشکی ایران، بزرگراه شهید همت غرب بین تقاطع شیخ فضل الله و شهید چمران، ستاد مرکزی، طبقه ۵، معاونت تحقیقات و فناوری با شماره ۸۶۷۰۲۵۳۰ تماس گرفته و مشکل خود را به صورت شفاهی یا کتبی مطرح نمایم.

۱۴. این فرم اطلاعات و رضایت آگاهانه در دو نسخه تنظیم شده و پس از امضا یک نسخه در اختیار من و نسخه دیگر در اختیار مجری قرار خواهد گرفت.

اینجانب موارد فوق‌الذکر را خواندم و فهمیدم و بر اساس آن رضایت آگاهانه خود را برای شرکت در این پژوهش اعلام می‌کنم.

امضای شرکت کننده

شماره تماس شرکت کننده

اینجانب دکتر مهرناز کجباف والا خود را ملزم به اجرای تعهدات مربوط به مجری در مفاد فوق دانسته و متعهد می‌گردم در تأمین حقوق و ایمنی شرکت کننده در این پژوهش تلاش نمایم.

مهر و امضای مجری پژوهش
