# Supplementary material for: The effects of aerobic exercises compared to conventional chest physiotherapy on pulmonary function, functional capacity, sputum culture, and quality of life in children and adolescents with cystic fibrosis: a study protocol for randomized controlled trial study
Source: Trials. 2023 Oct 28;24:695. doi: 10.1186/s13063-023-07719-w (PMC10612191; doi:10.1186/s13063-023-07719-w)
Supplement: Supplementary file 3 — Additional file 3. [file 13063_2023_7719_MOESM3_ESM.pdf]

پرسشنامه کیفیت زندگی فیبروز کیستیک – بازبینی شده

Cystic fibrosis questionnaire – revised

۱- پرسش نامه کیفیت زندگی فیبروز کیستیک در کودکان ۶ تا ۱۱ سال و ۱۲ تا ۱۳ سال

۱- شما توانستی به همان سرعت دیگران قدم بزنی؟

خیلی صحیح است اغلب صحیح است گاهی اوقات صحیح است هیچ وقت صحیح نیست

۲- شما قادر بودی به همان سرعت دیگران از پله ها بالا بروی؟

خیلی صحیح است اغلب صحیح است گاهی اوقات صحیح است هیچ وقت صحیح نیست

۳- شما قادر به دویدن، پریدن، بالارفتن همانطور که می خواستی بودی.

خیلی صحیح است اغلب صحیح است گاهی اوقات صحیح است هیچ وقت صحیح نیست

۴- شما قادر بودی با سرعت و تا زمانی مشابه با دیگران بدوی.

خیلی صحیح است اغلب صحیح است گاهی اوقات صحیح است هیچ وقت صحیح نیست

۵- شما قادر بودی در ورزش هایی که از آن لذت می بردی شرکت کنی.

خیلی صحیح است اغلب صحیح است گاهی اوقات صحیح است هیچ وقت صحیح نیست

۶- شما درحمل کردن یا بلند کردن چیزهای سنگین مثل کتاب، کیف مدرسه و یا کوله پشتی مشکل داشتی؟

خیلی صحیح است اغلب صحیح است گاهی اوقات صحیح است هیچ وقت صحیح نیست

در طی دوهفته گذشته به چه میزان:

۱- احساس خستگی داشتی؟

همیشه اغلب گاهی اوقات هرگز

۲- احساس دیوانگی (عصبانیت) داشتی؟

همیشه اغلب گاهی اوقات هرگز

۳- احساس ناراحتی (بد خلقی) کردی؟

همیشه اغلب گاهی اوقات هرگز

۴- احساس نگرانی داشتی؟

همیشه اغلب گاهی اوقات هرگز

۵- احساس غم کردی؟

همیشه اغلب گاهی اوقات هرگز

۶- در به خواب رفتن مشکل داشتی؟

همیشه اغلب گاهی اوقات هرگز

۷- رویاهای بد یا کابوس شبانه داشتی؟

همیشه اغلب گاهی اوقات هرگز

۸- احساس خوب درباره خودت داشتی؟

همیشه اغلب گاهی اوقات هرگز

۹- مشکل در غذا خوردن داشتی؟

همیشه اغلب گاهی اوقات هرگز

۱۰- مجبور بودی فعالیت های سرگرم کننده را برای انجام درمان های خود متوقف کنی؟

همیشه اغلب گاهی اوقات هرگز

۱۱- شما تحت فشار قرار گرفتی که بخوری؟

همیشه اغلب گاهی اوقات هرگز

در طی ۲ هفته گذشته:

۱- شما قادر بودی تمام درمان های خود را انجام دهی؟

خیلی صحیح است اغلب صحیح است گاهی اوقات صحیح است هیچ وقت صحیح نیست

۲- شما از غذا خوردن لذت بردی؟

خیلی صحیح است اغلب صحیح است گاهی اوقات صحیح است هیچ وقت صحیح نیست

۳- شما با دوستان زیادی دورهم جمع شدید؟

خیلی صحیح است اغلب صحیح است گاهی اوقات صحیح است هیچ وقت صحیح نیست

۴- بیشتر از آنچه می خواستی اغلب اوقات در خانه ماندی؟

خیلی صحیح است اغلب صحیح است گاهی اوقات صحیح است هیچ وقت صحیح نیست

۵- شما احساس راحتی درخوابیدن دور از خانه (در خانه دوستان فامیل و غیره) داشتی؟

خیلی صحیح است اغلب صحیح است گاهی اوقات صحیح است هیچ وقت صحیح نیست

۶- احساس کردی کنار گذاشته شدی؟

خیلی صحیح است اغلب صحیح است گاهی اوقات صحیح است هیچ وقت صحیح نیست

۷- شما اغلب دوستان را به منزلتان دعوت کردی؟

خیلی صحیح است اغلب صحیح است گاهی اوقات صحیح است هیچ وقت صحیح نیست

۸- شما مورد تمسخر سایر کودکان قرار گرفتی؟

خیلی صحیح است اغلب صحیح است گاهی اوقات صحیح است هیچ وقت صحیح نیست

۹- شما در بحث در مورد بیماریتان با سایرین (دوستان، معلمین) احساس راحتی داری؟

خیلی صحیح است اغلب صحیح است گاهی اوقات صحیح است هیچ وقت صحیح نیست

۱۰- شما فکر کردی که خیلی کوتاه هستی؟

خیلی صحیح است اغلب صحیح است گاهی اوقات صحیح است هیچ وقت صحیح نیست

۱۱- شما فکر کردی که خیلی لاغر هستی؟

خیلی صحیح است اغلب صحیح است گاهی اوقات صحیح است هیچ وقت صحیح نیست

۱۲- شما فکر کردی که با سایر همسن هایت از نظر فیزیکی تفاوت داری؟

خیلی صحیح است اغلب صحیح است گاهی اوقات صحیح است هیچ وقت صحیح نیست

۱۳- انجام دادن درمان هایت تو را آزار داد؟

خیلی صحیح است اغلب صحیح است گاهی اوقات صحیح است هیچ وقت صحیح نیست

چه میزان در طی دو هفته گذشته:

۱- در طی روز سرفه کردی؟

همیشه اغلب گاهی اوقات هرگز

۲- چون سرفه می کردی در طی شب بیدار شدی؟

همیشه اغلب گاهی اوقات هرگز

۳- مجبور بودی خلط را با سرفه بالا ببری؟

همیشه اغلب گاهی اوقات هرگز

۴- نفس کشیدن مشکل دار داشتی؟

همیشه اغلب گاهی اوقات هرگز

۵- معده شما درد داشت؟

همیشه اغلب گاهی اوقات هرگز

۲- پرسش نامه کیفیت زندگی فیبروز کیستیک مرتبط با والدین کودکان ۶ تا ۱۳ سال

به چه میزان کودک شما مشکل دارد در:

۱- انجام فعالیت های شدید مثل دویدن یا بازی های ورزشی:

دشواری زیاد مقداری دشواری کمی دشواری دشوار نبود

۲- راه رفتن به سرعت دیگران:

دشواری زیاد مقداری دشواری کمی دشواری دشوار نبود

۳- بالا رفتن از پله ها با همان سرعت دیگران:

دشواری زیاد مقداری دشواری کمی دشواری دشوار نبود

۴- حمل کردن یا بلند کردن چیزهای سنگین مثل کتاب، موادغذایی یا کیف مدرسه:

دشواری زیاد مقداری دشواری کمی دشواری دشوار نبود

۵- بالارفتن از چندین پلکان از پله ها :

دشواری زیاد مقداری دشواری کمی دشواری دشوار نبود

در طی دو هفته گذشته مشخص کنید چند نوبت کودک شما:

۱- خوشحال به نظر می رسید:

همیشه اغلب گاهی هرگز

۲- نگران به نظر می رسید:

همیشه اغلب گاهی هرگز

۳- خسته به نظر می رسید:

همیشه اغلب گاهی هرگز

۴- زود رنج به نظر می رسید:

همیشه اغلب گاهی هرگز

۵- خوب به نظر می رسید:

همیشه	اغلب	گاهی	هرگز

۶- بد خلق به نظر می رسید:

همیشه	اغلب	گاهی	هرگز

۷- پر انرژی به نظر می رسید:

همیشه	اغلب	گاهی	هرگز

۸- به علت بیماریش یا درمان هایش ازمدرسه یا سایر فعالیتها غایب بود یا دیر کرد:

همیشه	اغلب	گاهی	هرگز

۹- میزانی که کودک شما در ورزش ها یا فعالیت های فیزیکی شرکت داشت، مثل کلاس ورزش:

- در فعالیت های فیزیکی شرکت نداشته است.

-کمتر از معمول در ورزش ها شرکت داشته است.

- به همان اندازه معمول شرکت کرده ولی با کمی سختی.

- توانسته است در فعالیت های فیزیکی بدون هیچ سختی شرکت کند.

۱۰- میزانی که کودک شما در راه رفتن مشکل دارد:

- او می تواند مدت طولانی راه برود بدون اینکه خسته شود.

- او می تواند مدت طولانی راه برود ولی خسته می شود.

- او نمی تواند مدت طولانی راه برود چون به سرعت خسته می شود.

- او از راه رفتن در هر زمان ممکن اجتناب می کند چون این کار برایش بسیارخسته کننده است.

در مورد وضعیت سلامتی کودکتان در دو هفته گذشته فکر کنید، میزانی که جملات پایین برای کودک شما صحیح یا غلط است را نشان دهید:

۱- فرزند من در بهبودی بعد از تلاش فیزیکی مشکل دارد:

خیلی صحیح است　　قدری صحیح است　　قدری غلط است　　خیلی غلط است

۲- وعده های غذایی یک مبارزه است:

خیلی صحیح است　　قدری صحیح است　　قدری غلط است　　خیلی غلط است

۳- درمان های فرزند من در سر راه فعالیت های اوست.

خیلی صحیح است　　قدری صحیح است　　قدری غلط است　　خیلی غلط است

۴- فرزند من احساس میکند در مقایسه با سایر کودکان همسن کوچک است:

خیلی صحیح است　　قدری صحیح است　　قدری غلط است　　خیلی غلط است

۵- فرزند من احساس می کند تفاوت فیزیکی با سایر کودکان همسن دارد:

خیلی صحیح است　　قدری صحیح است　　قدری غلط است　　خیلی غلط است

۶- فرزند من فکر می کند او خیلی لاغر است:

خیلی صحیح است　　قدری صحیح است　　قدری غلط است　　خیلی غلط است

۷- فرزند من احساس سلامتی می کند:

خیلی صحیح است　　قدری صحیح است　　قدری غلط است　　خیلی غلط است

۸- فرزند من تمایل به کناره گیری (عقب نشینی) دارد:

خیلی صحیح است　　قدری صحیح است　　قدری غلط است　　خیلی غلط است

۹- فرزند من یک زندگی نرمال را اداره میکند:

خیلی صحیح است　　قدری صحیح است　　قدری غلط است　　خیلی غلط است

۱۰- فرزند من تفریح کمتری نسبت به معمول دارد:

خیلی صحیح است　　قدری صحیح است　　قدری غلط است　　خیلی غلط است

۱۱- فرزند من برای همراهی با دیگران مشکل دارد:

خیلی صحیح است قدری صحیح است قدری غلط است خیلی غلط است

۱۲- فرزند من در تمرکز کردن مشکل دارد:

خیلی صحیح است قدری صحیح است قدری غلط است خیلی غلط است

۱۳- فرزند من می تواند کار مدرسه یا فعالیت های تابستانه خود را ادامه دهد:

خیلی صحیح است قدری صحیح است قدری غلط است خیلی غلط است

۱۴- فرزند من در انجام دادن فعالیت های مدرسه یا تابستان به خوبی معمول نیست.

خیلی صحیح است قدری صحیح است قدری غلط است خیلی غلط است

۱۵- فرزند من زمان زیادی برای درمان هایش هر روز صرف می کند:

خیلی صحیح است قدری صحیح است قدری غلط است خیلی غلط است

۱۶- چقدر برای فرزند شما انجام دادن درمان هایش هر روزمشکل است:

هیچ وقت کمی اغلب خیلی

۱۷- فکر می کنید اکنون سلامتی فرزند شما چگونه است؟

خیلی خوب خوب متوسط ضعیف

در مورد سلامتی فرزندتان در طی دو هفته گذشته فکر کنید، چه میزان جملات زیر صحیح یا غلط است:

۱-فرزند من در گرفتن وزن مشکل داشت:

نظر خوبی است تاحدی کمی هرگز

۲- فرزند من احساس فشار در سینه خود داشت:

نظر خوبی است تاحدی کمی هرگز

۳- فرزند من در طی روز سرفه داشت:

نظر خوبی است تاحدی کمی هرگز

۴- فرزند من خلط را با سرفه بالا می آورد:

نظر خوبی است		تاحدی		کمی		هرگز

۵- خلط فرزند من اغلب :

- شفاف بود

- شفاف تا زرد

- زرد تا سبز

- سبز همراه با کمی خون

- نمی دانم

- سرفه همراه با بالا آرودن خلط نداشت

در طی دو هفته گذشته:

۱- فرزند من خس خس سینه داشت (ویز):

همیشه		اغلب		گاهی اوقات		هرگز

۲- فرزند من در نفس کشیدن مشکل داشت:

همیشه		اغلب		گاهی اوقات		هرگز

۳- فرزند من به علت سرفه کردن درطی شب بیدار شد:

همیشه		اغلب		گاهی اوقات		هرگز

۴- فرزند من گاز (نفخ) داشت:

همیشه		اغلب		گاهی اوقات		هرگز

۵- فرزند من اسهال داشت:

همیشه		اغلب		گاهی اوقات		هرگز

۶- فرزند من درد شکم داشت:

همیشه اغلب گاهی اوقات هرگز

۷- فرزند من مشکلات خوردن داشت:

همیشه اغلب گاهی اوقات هرگز

۳- پرسش نامه کیفیت زندگی فیبروز کیستیک در کودکان ۱۴ سال به بالا

در طی ۲ هفته گذشته به چه میزان مشکل داشتی در:

۱- انجام فعالیت های شدید مثل دویدن یا بازی های ورزشی:

دشواری زیاد　　مقداری دشواری　　کمی دشواری　　دشوار نبود

۲- راه رفتن به سرعت دیگران:

دشواری زیاد　　مقداری دشواری　　کمی دشواری　　دشوار نبود

۳- حمل کردن یا بلند کردن چیزهای سنگین مثل کتاب، موادغذایی یا کیف مدرسه:

دشواری زیاد　　مقداری دشواری　　کمی دشواری　　دشوار نبود

۴- بالارفتن از یک پلکان از پله ها :

دشواری زیاد　　مقداری دشواری　　کمی دشواری　　دشوار نبود

۵- بالا رفتن از پله ها با همان سرعت دیگران:

دشواری زیاد　　مقداری دشواری　　کمی دشواری　　دشوار نبود

در طی دو هفته گذشته:

۱- احساس خوبی داشتی؟

همیشه　　اغلب　　گاهی　　هرگز

۲- احساس نگرانی داشتی؟

همیشه　　اغلب　　گاهی　　هرگز

۳- احساس بی فایدگی کردی؟

همیشه　　اغلب　　گاهی　　هرگز

۴- احساس خستگی کردی؟

همیشه　　اغلب　　گاهی　　هرگز

۵- احساس کردی پر انرژی بودی؟

همیشه اغلب گاهی هرگز

۶- احساس خستگی شدید کردی؟

همیشه اغلب گاهی هرگز

۷- احساس غم کردی؟

همیشه اغلب گاهی هرگز

در مورد وضعیت سلامتی خود در دو هفته گذشته فکر کنید:

۱- در چه حدی راه رفتن مشکل دار داری؟

- تو می توانی برای زمان طولانی بدون اینکه خسته شوی راه بروی.

- تو می توانی برای زمان طولانی راه بروی ولی خسته می شوی.

- تو نمی توانی زمان طولانی راه بروی چون به سرعت خسته می شوی.

- تو از راه رفتن هر زمان امکان دارد امتناع می کنی چون برای تو بسیار خسته کننده است.

۲- تو چه احساسی درباره خوردن داری؟

- فقط فکر کردن درباره غذا احساس بیماری برایت ایجاد می کند.

- تو هرگز از غذا خوردن لذت نمی بری.

- تو اغلب قادر هستی از غذاخوردن لذت ببری.

- تو همیشه قادر هستی از غذاخوردن لذت ببری.

۳- تا چه حد انجام درمان هایت زندگی روزمره شما را دشوارتر کرده است؟

اصلا کمی نسبتا زیاد

۴- چه مدت زمانی از روز را در حال حاضر صرف درمان هایت می کنی؟

زیاد قدری کمی نه خیلی زیاد

۵- چه دشواری برای شما دارد که درمان هایت (شامل درمانهای دارویی) را هر روز انجام دهی؟

اصلا کمی نسبتا زیاد

۶- فکر می کنی الان سلامتی شما چطور هست؟

خیلی خوب خوب نامناسب ضعیف

در مورد وضعیت سلامت خود در طی دو هفته گذشته فکر کنید و بگویید به چه اندازه موارد پایین صحیح یا غلط است؟

۱- من بعد از فعالیت فیزیکی در بهبودی مجدد مشکل دارم.

خیلی صحیح است قدری صحیح است قدری غلط است خیلی غلط است

۲- من مجبور هستم فعالیت های شدید مثل دویدن یا بازی های ورزشی را محدود کنم.

خیلی صحیح است قدری صحیح است قدری غلط است خیلی غلط است

۳- من باید خودم را مجبور کنم که بخورم.

خیلی صحیح است قدری صحیح است قدری غلط است خیلی غلط است

۴- من مجبور هستم در خانه بیشتر از آنچه می خواهم بمانم.

خیلی صحیح است قدری صحیح است قدری غلط است خیلی غلط است

۵- من در بحث در مورد بیماریم با سایرین (دوستان، معلمین) احساس راحتی دارم.

خیلی صحیح است قدری صحیح است قدری غلط است خیلی غلط است

۶- من فکر میکنم خیلی لاغر هستم.

خیلی صحیح است قدری صحیح است قدری غلط است خیلی غلط است

۷- من فکر میکنم از سایر همسن هایم متفاوت به نظر می رسم.

خیلی صحیح است قدری صحیح است قدری غلط است خیلی غلط است

۸- من احساس بدی درباره ظاهر فیزیکم دارم.

خیلی صحیح است قدری صحیح است قدری غلط است خیلی غلط است

۹- مردم می ترسند که ممکن است مسری باشم.

خیلی صحیح است قدری صحیح است قدری غلط است خیلی غلط است

۱۰- من با دوستان زیادی دورهم جمع می شویم.

خیلی صحیح است قدری صحیح است قدری غلط است خیلی غلط است

۱۱- من فکر میکنم سرفه کردن من دیگران را آزار میدهد.

خیلی صحیح است قدری صحیح است قدری غلط است خیلی غلط است

۱۲- من احساس راحتی در رفتن بیرون در شب دارم.

خیلی صحیح است قدری صحیح است قدری غلط است خیلی غلط است

۱۳- من اغلب احساس تنهایی میکنم.

خیلی صحیح است قدری صحیح است قدری غلط است خیلی غلط است

۱۴- من احساس سلامت میکنم.

خیلی صحیح است قدری صحیح است قدری غلط است خیلی غلط است

۱۵- این دشوار است که برنامه ریزی هایی برای آینده کرد.

خیلی صحیح است قدری صحیح است قدری غلط است خیلی غلط است

۱۶- من زندگی نرمالی را رهبری میکنم.

خیلی صحیح است قدری صحیح است قدری غلط است خیلی غلط است

سوالات بعدی درباره کارهای مدرسه و یا سایر کارهای روزانه است.

۱- تا چه حد در ادامه کار مدرسه ات، کارحرفه ای یا سایر فعالیت های روزانه در ۲ هفته گذشته دچار مشکل شدی؟

- شما مشکلی در ادامه کار نداشتی.

- شما موفق شدی به کار خود ادامه دهی اما کار دشواری بوده است.

- شما پشت سر گذاشتی.

- شما اصلا قادر نبودی این فعالیت ها را انجام دهی.

۲- چندوقت در مدرسه یا کار غایب بودی یا ناتوانی در تکمیل فعالیت های روزانه در طی ۲ هفته گذشته به علت بیماریت یا درمانت داشتی؟

همیشه اغلب گاهی هرگز

۳- چند وقت سیستیک فیبروزیس جلوی ملاقات شما با مدرسه تان، کار یا اهداف شخصی شما را می گیرد؟

همیشه اغلب گاهی هرگز

۴- چند وقت سیستیک فیبروزیس جلوی بیرون رفتن از خانه برای اجرای مواردی مثل خریدکردن یا رفتن به بانک را می گیرد؟

همیشه اغلب گاهی هرگز

درباره احساس خود در دوهفته گذشته بگویید:

۱- در وزن گرفتن دچار مشکل شدی؟

خیلی گاهی اوقات کمی اصلا

۲- فشاری در قفسه سینه خود داشته ای؟

خیلی گاهی اوقات کمی اصلا

۳- در طی روز سرفه کرده ای؟

خیلی	گاهی اوقات	کمی	اصلا

۴- آیا مجبور شده ای خلط را با سرفه بالا آوری؟

خیلی	گاهی اوقات	کمی	اصلا

۵- خلط شما بیشتر اوقات:

- شفاف
- شفاف تا زرد
- زرد تا سبز
- سبز همراه با خون کم
- نمی دانم
- من سرفه خلط دار ندارم

چند وقت در طی دو هفته گذشته:

۱- آیا ویزینگ داشته ای؟

همیشه	اغلب	گاهی اوقات	هرگز

۲- آیا تنفس مشکل داشته ای؟

همیشه	اغلب	گاهی اوقات	هرگز

۳- آیا مجبور شدی در طی شب به علت اینکه سرفه داشتی بیدارشوی؟

همیشه	اغلب	گاهی اوقات	هرگز

۴- آیا مشکلاتی با گاز (نفخ شکم) داشته ای؟

همیشه	اغلب	گاهی اوقات	هرگز

۵- آیا اسهال داشته ای؟

همیشه اغلب گاهی اوقات هرگز

۶- آیا درد شکمی داشته ای؟

همیشه اغلب گاهی اوقات هرگز

۷- آیا مشکلات خوردن داشته ای؟

همیشه اغلب گاهی اوقات هرگز
