# Supplementary material for: The effects of aerobic exercises compared to conventional chest physiotherapy on pulmonary function, functional capacity, sputum culture, and quality of life in children and adolescents with cystic fibrosis: a study protocol for randomized controlled trial study
Source: Trials. 2023 Oct 28;24:695. doi: 10.1186/s13063-023-07719-w (PMC10612191; doi:10.1186/s13063-023-07719-w)
Supplement: Supplementary file 4 — Additional file 4. [file 13063_2023_7719_MOESM4_ESM.pdf]

به نام خدا

پرسشنامه عوارض احتمالی

نام و نام خانوادگی: تاریخ:

آدرس و شماره تلفن: امضاء:

لطفا مواردی را که در طی ۶هفته مطالعه برای شما اتفاق افتاده است، مشخص نموده و اگر تعداد تقریبی تکرار آنها را به خاطر دارید، بنویسید.

☐۱- گریه و بی قراری در طی جلسه درمان (بسیار زیاد/ زیاد/ متوسط/ کم/ هیچگاه)

☐۲- تشدید سرفه ها (بسیار زیاد/ زیاد/ متوسط/ کم/ هیچگاه)

☐۳- احساس درد یا سوزش در قفسه سینه و دنده ها (بسیار زیاد/ زیاد/ متوسط/ کم/ هیچگاه)

☐۴- تهوع و استفراغ (بسیار زیاد/ زیاد/ متوسط/ کم/ هیچگاه)

☐۵- تخلیه خلط خونی (بسیار زیاد/ زیاد/ متوسط/ کم/ هیچگاه)

☐۶- احساس فشار و سنگینی در قفسه سینه (بسیار زیاد/ زیاد/ متوسط/ کم/ هیچگاه)

☐۷- تنگی نفس و دشواری تنفس (بسیار زیاد/ زیاد/ متوسط/ کم/ هیچگاه)

☐۸- وقوع ریفلاکس یا بازگشت محتویات معده به مری (بسیار زیاد/ زیاد/ متوسط/ کم/ هیچگاه)

☐۹- احساس سرگیجه (بسیار زیاد/ زیاد/ متوسط/ کم/ هیچگاه)

☐۱۰- احساس ضعف و خستگی شدید (بسیار زیاد/ زیاد/ متوسط/ کم/ هیچگاه)

☐۱۱- افت قند خون درصورتیکه چک شده باشد (بسیار زیاد/ زیاد/ متوسط/ کم/ هیچگاه)

☐۱۲- احساس درد، کوفتگی و گرفتگی در عضلات پاها (بسیار زیاد/ زیاد/ متوسط/ کم/ هیچگاه)

☐۱۳- احساس تپش قلب (بسیار زیاد/ زیاد/ متوسط/ کم/ هیچگاه)

۱۴- سایر موارد: ...
